# A retrospective study on the incidence of acute kidney injury and its early prediction using troponin-I in cooled asphyxiated neonates

**DOI:** 10.1038/s41598-020-72717-w

**Published:** 2020-09-24

**Authors:** Tze Yee Diane Mok, Min-Hua Tseng, Jin-Chiao Lee, Yu-Ching Chou, Reyin Lien, Mei-Yin Lai, Chien-Chung Lee, Jainn-Jim Lin, I-Jun Chou, Kuang-Lin Lin, Ming-Chou Chiang

**Affiliations:** 1 Department of Pediatrics, New Taipei Municipal TuCheng Hospital, New Taipei, Taiwan; 2 Division of Neonatology, Department of Pediatrics, Chang Gung Memorial Hospital, Chang Gung University College of Medicine, No. 5, Fushing St., Guishan Dist., Taoyuan City 333, Taiwan; 3 Division of Pediatric Nephrology, Department of Pediatrics, Chang Gung Memorial Hospital, Chang Gung University College of Medicine, Taoyuan, Taiwan; 4 Division of Liver and Transplantation Surgery, Department of General Surgery, Chang Gung Memorial Hospital, Chang Gung University College of Medicine, Taoyuan, Taiwan; 5 School of Public Health, National Defense Medical Center, Taipei, Taiwan; 6 Division of Respiratory Therapy, Chang Gung Memorial Hospital, Chang Gung University College of Medicine, Taoyuan, Taiwan; 7 Graduate Institute of Clinical Medical Sciences, Chang Gung University College of Medicine, Taoyuan, Taiwan; 8 Division of Pediatric Critical Care Medicine and Pediatric Neurocritical Care Center, Chang Gung Memorial Hospital, Chang Gung University College of Medicine, Taoyuan, Taiwan; 9 Division of Pediatric Neurology, Department of Pediatrics, Chang Gung Memorial Hospital, Chang Gung University College of Medicine, Taoyuan, Taiwan; 10 Study Group of Intensive and Integrated Care for Pediatric Central Nervous System (iCNS Group), Taoyuan, Taiwan

**Keywords:** Biomarkers, Diseases, Medical research, Nephrology

## Abstract

Acute kidney injury (AKI) is a common complication of perinatal asphyxia and is associated with poorer short-term and long-term outcomes. This retrospective study describes the incidence of AKI in asphyxiated neonates who have received therapeutic hypothermia using the proposed modified Kidney Diseases: Improving Global Outcomes (KDIGO) definition and investigates clinical markers that would allow earlier recognition of at-risk neonates. We included asphyxiated  neonates who underwent therapeutic hypothermia between the period of January 2011 and May 2018 in our study. The serum creatinine levels within a week of birth were used in establishing AKI according to the modified KDIGO definition. Demographic data, resuscitation details, laboratory results and use of medications were collected and compared between the AKI and non-AKI groups to identify variables that differed significantly. A total of 66 neonates were included and 23 out of them (35%) were found to have AKI. The neonates with AKI had a lower gestational age (*p* = 0.006), lower hemoglobin level (*p* = 0.012), higher lactate level before and after therapeutic hypothermia (*p* = 0.013 and 0.03 respectively) and higher troponin-I level after therapeutic hypothermia (*p* < 0.001). After logistic regression analysis, elevated troponin-I after therapeutic hypothermia was independently associated with risk of AKI (OR 1.69, 95% CI 1.067–2.699, *p* = 0.025). The receiver operating curve showed that troponin-I after therapeutic hypothermia had an area under curve of 0.858 at the level 0.288 ng/ml. Our study concludes that the incidence of AKI among asphyxiated newborns who received therapeutic hypothermia is 35% and an elevated troponin-I level after therapeutic hypothermia is independently associated with an increased risk of AKI in asphyxiated newborns.

## Introduction

In recent years, there has been a substantial advancement in the study of neonatal acute kidney injury (AKI). It has become apparent that although serum creatinine levels return to normal after AKI episodes, they are associated with increased risk of mortality, increased length of hospital stay and chronic kidney disease^1^. Neonates with perinatal asphyxia are at risk of AKI as redistribution of cardiac output occurs during hypoxia to maintain cerebral, cardiac and adrenal perfusion, thereby reducing oxygen supply to the kidneys^2^. The renal parenchymal cells also have a limited capacity for anaerobic respiration and are highly susceptible to reperfusion injury^3^.

In the past, studies on AKI in neonates with perinatal asphyxia have reported variable incidence, ranging from 11.7 to 60% based on different diagnostic criteria used^4,5^. In 2013, a standardized definition of AKI termed the neonatal modified Kidney Diseases: Improving Global Outcomes (KDIGO) was proposed to allow for consistency throughout studies and it stages AKI using absolute rise of serum creatinine (SCr) from a previous trough or the decrease of urine output over certain time frames^6,7^. Past studies have reported that the renal failure in asphyxiated neonates is often non-oliguric, with many neonates maintaining a urine output of more than 1 ml/kg/h despite significant renal dysfunction^8^. Thus, more recent studies have adopted a SCr based approach in defining neonatal AKI. However, using the changes in SCr for the diagnosis of AKI has its limitations, including the presence of maternal creatinine and the delayed rise of SCr following an insult^9,10^. When it comes to AKI, the earlier the recognition, the higher the chances of survival as implementation of preventive strategies and treatment of anticipated complications can be carried out.

In this retrospective study, the neonatal modified KDIGO criteria was used to define AKI. The aim of the study is to describe the incidence of AKI and discover the clinical markers associated with AKI in asphyxiated neonates treated with therapeutic hypothermia.

## Results

**Figure 1:**
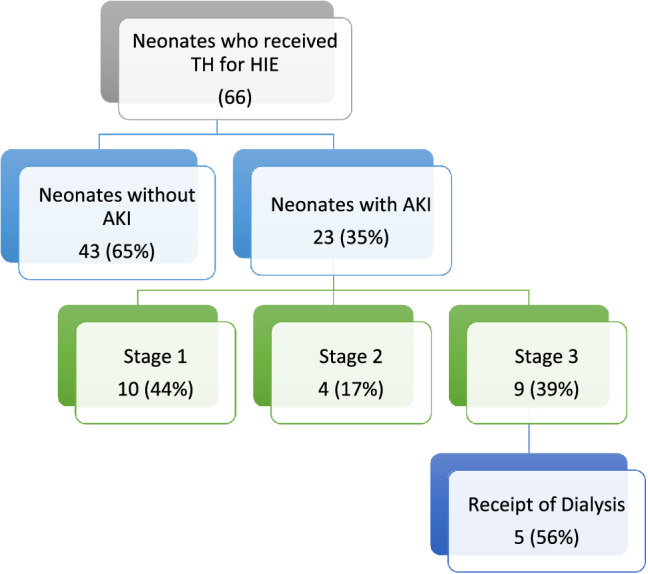
Flow chart of enrolled patients. The 66 patients enrolled were classified into the AKI group and non-AKI group while those in the AKI group were further stratified into three stages based on the modified KDIGO definition. The three stages correlate with increasing severity. Receipts of dialysis are considered to have stage III AKI regardless of their serum creatinine levels. *TH* therapeutic hypothermia, *HIE* hypoxic ischemic encephalopathy, *AKI* acute kidney injury.

**Figure 2:**
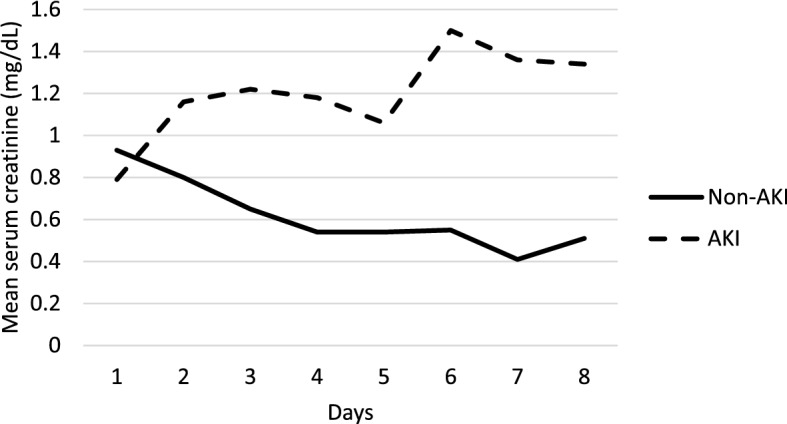
Trends of serum creatinine levels within one week of life. A comparison between the trend of mean serum creatinine values in the AKI group and non-AKI group within the first week of birth. *AKI* acute kidney injury.

A total of 66 neonates who underwent 72 hours of therapeutic hypothermia for perinatal asphyxia and HIE were included in our study. As part of our standardized protocol, all neonates had SCr levels measured before cooling, at 24-h, 48-h and 78–80 h. 85% (56/66) of the neonates had additional one or more SCr measurement within the first week of birth. Based on the neonatal modified KDIGO criteria, 35% (23/66) were found to have AKI. Among the neonates with AKI, 10 were classified as stage one, 4 as stage two and 9 as stage three. 5 out of 9 neonates with stage three AKI were receipts of dialysis (Fig. 1). The trend of SCr levels in the AKI group and non-AKI group is presented in Fig. 2. 43% (10/23) of patients in the AKI group were diagnosed based on the rise of SCr that occurred within 72-h while the rest (57%) were not diagnosed until a rise in SCr occurred after 72-h.

The baseline parameters between the AKI-group and non-AKI group are shown in Table 1. The parameters found to be significantly different between the two groups include the median gestational age (37 weeks vs 39 weeks, *p* = 0.006), initial hemoglobin level (11.8 g/dl vs 15.7 g/dl, *p* = 0.012), lactate levels before hypothermia therapy (171.1 mg/dl vs 81.4 mg/dl, *p* = 0.013), lactate levels after hypothermia therapy (31.4 mg/dl vs 15.1 mg/dl, *p* = 0.003) and troponin-I levels after hypothermia therapy (0.47 ng/ml vs 0.07 ng/ml, *p* < 0.001).

**Table 1:** Comparison of patients’ baseline characteristics in the acute kidney injury and non-acute kidney injury group.

		AKI (n = 23)	Non-AKI (n = 43)	p value
Demographic data				
GA (weeks)	37 [37–38]		39 [37–40]	0.006
BBW (g)	2,865 [2399–3393]		2,969 [2705–3281]	0.500
Gender (male)	12(52%)		23(53%)	0.862
Apgar score at 1′	1 [0–5]		2 [0–4]	0.670
Apgar score at 5′	4 [1–6]		4 [2–6]	0.350
CPR at birth, n (%)	15 (65%)		26 (60%)	0.542
Outborn, n (%)	20 (87%)		34 (79%)	0.176
Laboratory data				
ABG (first)				
pH	7.01 [6.88–7.18]		7.16 [7.05–7.27]	0.120
PCO_2_, mmHg	32.0 [21.4–46.6]		30.5 [20.1–39.9]	0.791
PaO_2_, mmHg	84.9 [64.5–156.2]		108.5 [75.4–146.9]	0.931
HCO_3_, mm/l	7.9 [4.8–12.6]		11.1 [7.27–14.9]	0.181
Hemoglobin, g/dl	11.8 ± 5.2		15.7 ± 3.5	0.012
Lactate (before TH), mg/dl	171.1[11.9–318.4]		81.4[14.6–255.4]	0.013
Lactate (after TH), mg/dl	31.4 [18.7–77.6]		15.1 [11.5–28.4]	0.030
Troponin-I (before TH), ng/ml	0.34 [0.18–1.20]		0.14 [0.07–0.41]	0.600
Troponin-I (after TH), ng/ml	0.47 [0.17–1.86]		0.07 [0.04–0.16]	< 0.001
CPK, U/l	1771 [905–3818]		1,458 [779–2345]	0.299
CK-BB, %	12.40 [4.20–35.70]		11.20 [3.45–26.30]	0.817
CK-MB, %	4.10 [2.65–6.35]		4.26 [3.02–5.57]	0.861
CK-MM, %	76.90 [59.25–91.00]		81.85 [67.32–91.80]	0.445
Medications				
Inotropes, n (%)	20 (87%)		42 (97%)	0.460
Aminoglycosides, n (%)	6 (26%)		16 (37%)	0.460

*AKI* acute kidney injury, *GA* gestational age, *BBW* birth body weight, *CPR* cardiopulmonary resuscitation, *ABG* arterial blood gas, *TH* therapeutic hypothermia, *CPK* creatine phosphokinase, *CK-BB* creatine kinase brain band, *CK-MB* creatine kinase myocardial band, *CK-MM* creatine kinase muscle type.

**Figure 3:**
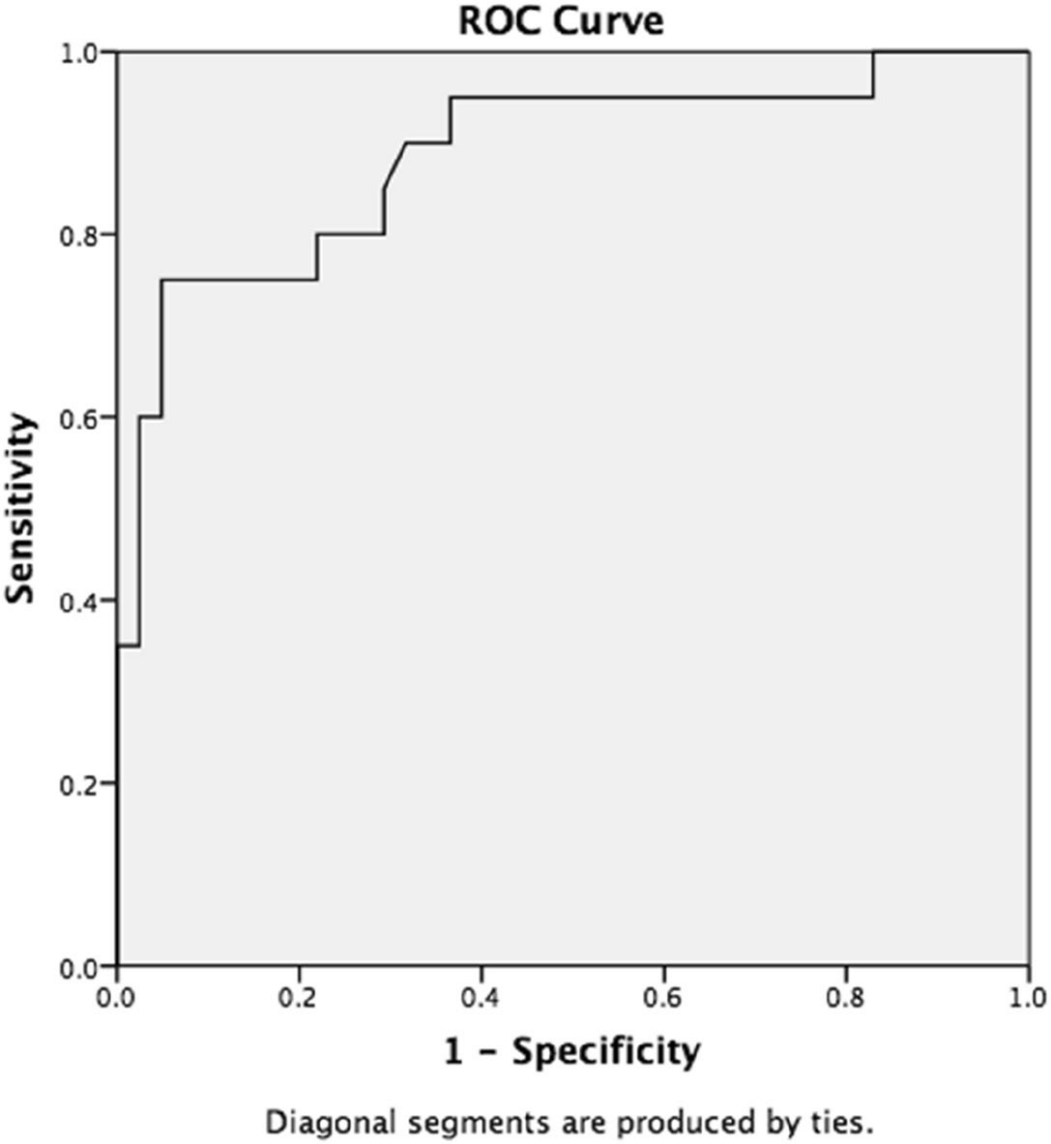
Receiver operating characteristic curve of troponin-I at 72-h in predicting acute kidney injury. The receiver operating curve (ROC) indicated that a cut-off value of troponin-I after therapeutic hypothermia was 0.288 ng/ml with 71% sensitivity and 95% specificity for predicting AKI in asphyxiated neonates. *AKI* acute kidney injury, *ROC* receiver operating curve.

On multivariate linear regression analysis, troponin-I level after hypothermia was found to be independently associated with the occurrence of AKI (OR 1.697, 95% CI 1.06–2.69, *p* = 0.025) (Table 2). To identify the level of troponin-I after therapeutic hypothermia that would best predict AKI, we plotted a receiver operating curve (ROC) as shown in Fig. 3. The ROC indicated that at a cut-off level of troponin-I after therapeutic hypothermia of 0.288 ng/ml, the area under curve (AUC) was 0.858. We rounded up 0.288 ng/ml to 0.3 ng/ml, which is also the reference value for a normal troponin-I level. The high troponin-I after therapeutic hypothermia (> 0.3 ng/ml) remained as an independent risk factor for AKI (OR 58.1, 95% CI 5.26–641.62, *p* = 0.001).

**Table 2:** Logistic regression analysis of clinical markers indicative of acute kidney injury.

	Univariate			Multivariate		
	OR	(95% CI)	p value	OR	(95% CI)	p value
GA	0.74	0.57–0.97	0.006	1.02	0.66–1.59	0.902
Hemoglobin	0.83	0.73–0.95	0.012	0.70	0.23–2.06	0.521
Lactate (before TH)	1.01	1.00–1.02	0.013	0.99	0.98–1.01	0.919
Lactate (after TH)	1.02	1.00–1.03	0.003	1.00	0.98–1.02	0.904
Tr-I (after TH) (0.1 ng/ml)	1.00	0.99–1.02	< 0.001	1.69	1.06–2.69	0.025

*GA* gestational age, *TH* therapeutic hypothermia, *Tr-I* troponin-I, *hr* hour, *OR* odds ratio, *CI* confidence interval.

## Discussion

In our study, the incidence of AKI among asphyxiated neonates receiving therapeutic hypothermia is 35%. Our study reveals that there were 13 (57%) patients whose diagnosis of AKI was made after 72-h of age. Although certain characteristics including a lower gestational age, lower hemoglobin level at birth, higher lactate before and after therapeutic hypothermia and higher troponin-I after therapeutic hypothermia appeared to be statistically significant on our initial univariate analysis, only a higher troponin-I level after therapeutic hypothermia stood out to be independently associated with AKI, especially at level > 0.3 ng/ml. This is the first study to associate a common biochemical marker, troponin-I, with the occurrence of AKI in asphyxiated neonates undergoing therapeutic hypothermia.

In the past, comparison of studies on neonatal AKI among asphyxiated neonates was difficult as different diagnostic criteria were used. Definitions frequently used included using an absolute SCr > 1.5 mg/dl, the pediatric Risk, Injury, Failure, Loss of kidney function, and End-stage kidney disease (pRIFLE) and Acute Kidney Injury Network (AKIN)^12–15^. In 2013, the neonatal modified KDIGO criteria was proposed as a standardized definition to allow for consistency throughout studies^7^. A number of studies on AKI in very low birth weight infants (VLBW) have utilized this definition^16,17^ but only two studies on AKI in asphyxiated neonates have used the neonatal modified KDIGO definition. Both Selewski et al. and Sarkar et al. investigated the incidence of AKI in asphyxiated neonates who underwent therapeutic hypothermia and have reported an incidence of 38% and 39% respectively using this definition^18,19^. The findings of these studies are consistent with the results of our study, reporting an incidence of 35% AKI among neonates with perinatal asphyxia who undergo therapeutic hypothermia.

The various definitions used to define AKI in neonates, including the neonatal modified KDIGO criteria are based on serum creatinine or urine output. Most studies on neonatal AKI have used SCr and its changes as the sole criteria for AKI. However, using a creatinine-based definition has its limitations. SCr is a marker of kidney function and not damage. Thus, a delay of 48–72 h rise of SCr is observed after an insult and about 50% of glomerular filtration rate has to be lost before an increase in SCr is evident^20,21^. This has led to intensification of research on biomarkers that would allow for earlier identification of AKI. Novel biomarkers that have shown promising results includes the urine neutrophil gelatinase-associated lipocalin, cystatin-c, kidney injury molecule-1, and interleukin-18^22^. However, these biomarkers are not available in most settings. The results of our study show that the level of cardiac troponin-I after 72-h of therapeutic hypothermia can help clinicians identify those at risk of AKI with a delayed rise in SCr.

The cardiac troponin-I is a specific cellular marker released by cardiomyocytes during cardiac injury. In neonates with perinatal asphyxia, it is a well-established marker for detection of myocardial ischemic changes and its levels correlate with the severity of hypoxic ischemic insult^23–25^. A randomized controlled trial by Rakesh et al. found that the troponin-I level decreased significantly in asphyxiated neonates who underwent hypothermia when compared with the normothermia group^26^. Another study by Liu et al. also suggested that hypothermia was cardio-protective in neonates with perinatal asphyxia, observing a significant reduction of cardiac troponin-I release following the therapeutic hypothermia^27^. In our study, this cardio-protective effect was observed in the non-AKI group, with the median troponin-I decreasing to normal after 72-h of therapeutic hypothermia while that in the AKI group remained high at 0.47 ng/ml.

The relationship between troponin-I and acute kidney injury in asphyxiated neonates can be explained by two possible mechanisms. First the higher level of troponin-I in the AKI group before and after therapeutic hypothermia could be due to a more severe degree myocardial injury, leading to decreased perfusion of the kidneys. This is also reflected in the higher lactate levels both before and after therapeutic hypothermia in the AKI group. Secondly, the impaired renal function in the AKI group could affect the excretion of troponin-I, causing the levels to remain high despite receiving therapeutic hypothermia. In adults, a study by Song et al. found that cardiac troponins including troponin-I were found to be elevated in subjects with AKI. In this study, patients with conditions known to cause elevated troponins were excluded, suggesting that impaired renal function during AKI is sufficient to influence the clearance of plasma troponins^28^. To date, there has been no studies in neonates linking AKI with the level of cardiac troponin-I.

Our study showed that with a cut-off value of troponin-I at 0.3 ng/ml after therapeutic hypothermia, the ROC yielded an AUC of 0.858 indicating troponin-I as good predictive tool for AKI at this value. This finding is important as it could provide clinician with the first clue of AKI in more than 50% of the asphyxiated neonates undergoing therapeutic hypothermia. With earlier recognition of AKI, management strategies including judicious use of fluids and diuretics, correction of acid–base and electrolyte imbalance, avoidance of nephrotoxic drugs could prevent further injury to the poorly functioning kidney.

Despite the significant findings of our study, we acknowledge that there are important limitations. Due to its retrospective design, the SCr data used for diagnosis of AKI was based on our standardized protocol for asphyxiated neonates undergoing therapeutic hypothermia. According to our therapeutic hypothermia protocol, the troponin-I is measured only before and after therapeutic hypothermia. Thus, whether a 24-h or 48-h troponin-I would allow for earlier prediction of AKI is a question yet to be answered by future prospective studies. Although limited by our small sample size, we report an incidence of AKI in asphyxiated neonates undergoing therapeutic hypothermia which is similar to the only two other studies that have used the recommended neonatal modified KDIGO criteria. We also highlight the use troponin-I level following therapeutic hypothermia to predict AKI. In patients whose rise in SCr is delayed beyond 72-h, this can be helpful to clinicians in making clinical judgements and thereby improving the outcome of these patients.

## Conclusion

The incidence of AKI among asphyxiated newborns who received therapeutic hypothermia is 35%. An elevated troponin-I level after therapeutic hypothermia is independently associated with an increased risk of AKI in asphyxiated newborns.

## Methods

### Enrolled patients

This retrospective study was approved by the ethics committee of Chang Gung Medical Hospital and waived the requirement to obtain informed consent of collecting anonymized data (IRB 201900607B0). All methods were carried out in accordance with relevant guidelines and regulations**.** Neonates with perinatal asphyxia and hypoxic ischemic encephalopathy (HIE) admitted to the neonatal intensive care unit (NICU) of Chang Gung Memorial Hospital, between the period of January 2011 and May 2018, were identified via a retrospective review of medical charts. Those who completed 72-h of therapeutic hypothermia were enrolled in this study. Neonates transferred from outside institutions were also included if they arrived within 6 hours of birth and were eligible for therapeutic hypothermia. In our institute, the patients who undergo therapeutic hypothermia must meet the following criteria: (A) neonates who are born at gestational age 36 weeks and above (B) initiation of therapeutic hypothermia within 6 hours of birth (C) evidence of moderate-to-severe encephalopathy and one of the following conditions: (1) severe acidosis (pH 7.00 or base deficit ≥ 16 mmol/l) within 1-h after birth, either from the umbilical cord or arterial or venous samples; (2) Apgar score 5 at 10 min; or (3) resuscitation ≥ 10 min after birth^11^.

### Therapeutic hypothermia protocol

Once the patients are deemed suitable for therapeutic hypothermia, we use whole-body cooling to induce therapeutic hypothermia. An esophageal probe is used for the measurement of core body temperature. The body temperature is measured every half an hour during the first 4-h, every hour between the 4th to 12th hour and every two hours thereafter. The hypothermic status is continued for 72-h with a targeted core temperature at 33–34 °C. The rewarming is then performed slowly with a core temperature rise of ≤ 0.5 °C per hour. Laboratory tests like hemogram, electrolytes, liver transaminases, blood urea, SCr, blood gas analyses are obtained before the initiation of hypothermia and then at 24-h, 48-h and after the rewarming process is completed which is around 78–80 h. After that, these laboratory tests are obtained at the discretion of the attending physician in charge. Lactate, troponin-I, creatine kinase (CPK) isoenzymes and ammonia levels are obtained before and after therapeutic hypothermia. Ventilator strategies are aimed at avoiding hyperoxemia and hypocapnia. Mean arterial blood pressure is maintained within the critical range of 40–60 mmHg and vasopressors are used when indicated. Fluid is normally restricted at 60–80 ml/kg/day. Empirical antibiotics are administered routinely upon admission and the most common regimens are Ampicillin and Gentamicin or Ampicillin and Cefotaxime. Amplitude-integrated electroencephalography (EEG) and/or continuous video-EEG are used to monitor brain activity. Anti-epileptic drugs, phenobarbital or levetiracetam, are given if clinical or subclinical seizures are observed.

### Data collection and statistical analysis

The clinical data of patients including gestational age, birth weight, Apgar scores, resuscitation details were recorded. Laboratory data and use of medications including antibiotics and vasopressors were also documented. Changes of SCr within the first week of life was used to establish a diagnosis of AKI based on the neonatal modified KDIGO criteria. Stage 1 AKI is defined as increase in SCr of more than 0.3 mg/dl within 48-h or 150–200% from baseline. Stage 2 AKI is defined as increase in SCr of more than 200–290% from baseline. Stage 3 AKI is defined as increase in SCr of more than 300% from baseline or SCr > 2.5 mg/dl or receipt of dialysis. The baseline SCr is the lowest previous SCr within 7 days. In our institute, the serum creatinine is measured using the colorimetric method Jaffe reaction.

The collected data were compared between the AKI group and non-AKI group to identify variables that differed significantly. Categorical variables were analyzed using Chi-square and reported as absolute numbers and percentage. Analysis of continuous variables were performed using both parametric and nonparametric procedures as appropriate. Data are reported as mean and standard deviation (SD) for continuous parametric data and as median with interquartile range for nonparametric data. Variables identified as being clinically significant for the development of AKI on univariate analysis were retained for multivariate analysis. A two-sided *p* value of ≤ 0.05 was considered significant. Statistical analyses were performed using SPSS, version 20.0 (IBM SPSS, Chicago, Illinois).

## Data Availability

The datasets generated during and/or analyzed during the current study are available from the corresponding author, Ming-Chou Chiang on reasonable request.
